# Transdiagnostic Dimensions towards Personality Pathology and Childhood Traumatic Experience in a Clinical Sample: Subtype Classification by a Cross-sectional Analysis

**DOI:** 10.1038/s41598-019-47754-9

**Published:** 2019-08-02

**Authors:** JunJie Wang, Wei Sun, XiaoChen Tang, LiHua Xu, YanYan Wei, HuiRu Cui, YingYing Tang, Li Hui, QiuFang Jia, Hongliang Zhu, JiJun Wang, TianHong Zhang

**Affiliations:** 1 Institute of Mental Health, Suzhou Guangji Hospital, The Affiliated Guangji Hospital of Soochow Unversity, Soochow Unversity, Suzhou, Jiangsu 215137 China; 2 Department of Neurosurgery, Pu Nan Hospital, Shanghai, 200125 China; 3 Shanghai Mental Health Center, Shanghai Jiaotong University School of Medicine, Shanghai Key Laboratory of Psychotic Disorders, Shanghai, 200030 P.R. China; 4 Bio-X Institutes, Key Laboratory for the Genetics of Developmental and Neuropsychiatric Disorders (Ministry of Education), Shanghai, P.R. China; 5 Brain Science and Technology Research Center, Shanghai Jiao Tong University, Shanghai, China

**Keywords:** Psychiatric disorders, Diagnosis

## Abstract

Psychiatric disorders are highly heterogeneous syndromes often explained by underlying and internalized personality disorder(PD) traits that are affected by externalized childhood trauma experiences(CTE). The present study investigated the differential subtype model by examining the association between PD traits and CTE in a clinical sample with transdiagnostic psychopathology. Outpatients(n = 2090) presenting for psychiatric treatment completed self-reported measures of PD traits(Personality Diagnostic Questionnaire) and the childhood adversity(Child Trauma Questionnaire). Canonical variates were generated by canonical correlation analysis(CCA) and then used for hierarchical cluster analysis to produce subtypes. A support vector machine(SVM) model was used and validated using a linear kernel to assess the utility of the extracted subtypes of outpatients in clinical diagnosis classifications. The CCA determined two linear combinations: emotional abuse related dissociality PD traits(antisocial and paranoid PD) and emotional neglect related sociality PD traits(schizoid, passive-aggressive, depressive, histrionic, and avoidant PD). A cluster analysis revealed three subtypes defined by distinct and relatively homogeneous patterns along two dimensions, and comprising 17.5%(cluster-1, n = 365), 34.8%(cluster-2, n = 727), and 47.8%(cluster-3, n = 998) of the sample, each with distinctive features of PD traits and CTE. These subtypes suggest more distinct PD trait correlates of CTE manifestations than were captured by clinical phenomenological diagnostic definitions. Our results highlight important subtypes of psychiatric patients that highlight PD traits and CTE that transcend current diagnostic boundaries. The three different subtypes reflect significant differences in PD and CTE characteristics and lend support to efforts to develop PD and childhood trauma targeted psychotherapy that extends to clinical diagnosis-based interventions.

## Introduction

Psychiatric disorder classifications are transformed by the enhanced knowledge of the RDoC (Research Domain Criteria) method^1,2^. Statistical modelling of complex clinical and behavioural datasets can facilitate the redefinition and reconceptualization of various diagnoses with unclear boundaries^3^. There is overlap in clinical manifestations and illness trajectories in major psychiatric categories such as affective disorders^4^, anxiety disorders^5,6^ and psychosis^7^. In our previous report^8^, we found that personality disorder (PD) was very common in psychiatric patients, and comorbidity between PD and other psychiatric disorders do not have fixed schema and are typically uncertainty. For instance, the proportions of anxiety disorders with co-morbid PD were varied from 2.1% (Schizotypal PD) to 13.4% (Obsessive–compulsive PD). The inverse is also true that the proportions of PD with co-morbid other psychiatric disorders were highly varied. The categorical methods for current psychiatric diseases has been highlighted as having significant problems such as arbitrary diagnostic thresholds and extensive overlap among diagnostic categories. Internationally, major transformations of psychiatric diagnoses from categorical to dimensional approaches are underway. In other ways, there are large quantities of evidence in the classification of psychiatric disorders with remarkably similar personality pathologies into distinct disorders^9,10^. Therefore, we assume that personality pathologies (PD traits) through dimensional approach rather than categorical diagnoses of PD may better reflect different subgroups of psychiatric patients.

Not only are personality pathologies associated with mental illness, but childhood traumatic experiences (CTE) are also closely related to it^11^. More crucial is that the relationship between CTE and PD is extremely close, possibly together, they affected subsequent psychiatric disorders^12,13^. However, PD classifications remain remarkably heterogeneous and are often associated with multiple forms of childhood traumatic experiences (CTE)^14^. Previous studies^12,13^ have tended to focus on the correlational pattern between PD and CTE, but have not yet tended to use these correlation characteristics to construct subtypes of mental disorders or subgroups of psychiatric patients. The present study applied, for the first time, a canonical correlation analysis (CCA) method for defining subtypes by clustering psychiatric patients, with different clinical diagnoses, according to the patterns of the relationship (canonical variates) between PD traits and CTE. We further tested whether the CCA-derived subtypes differed with regards to demographics, clinical classifications, and PD categories.

## Methods

### Subjects, settings and procedures

The study was conducted following the tenets of the Helsinki Declaration and approved by the Research Ethics Committee of the Shanghai Mental Health Center (SMHC). All participants gave written informed consent at the recruitment stage of the study. The sample consisted of 3,075 consecutive outpatients who visited psychiatric or psychological health services at SMHC in 2006, the largest clinical setting for psychiatric services which served more than 800,000 outpatients in 2018. The SMHC is not a regional psychiatric hospital, but serves patients from the whole country. There are two outpatient departments settings: psychiatric and psychocounseling units, which the former targeted patients with severe mental disorders (such as psychosis) and the later targeted for mental health problems (such as anxiety and depression).

In this survey, every 10^th^ subject in psychocounseling and every 20^th^ subject in psychiatric clinics was selected. Inclusion criteria were as follows: (i) age, 18–60 years; (ii) individuals with the capacity to provide informed consent; and (iii) an educational background of at least junior middle school. Junior middle school is part of nine-year compulsory education in China, which is a very low level of educational requirement. Patients with severe somatic diseases, acute phase of psychoses, and diagnoses of mental retardation or dementia were excluded. Details of the study procedures; study setting; and measurements and assessments, including the steps taken to ensure a very high quality control and assurance of the procedures, are reported elsewhere^8,15^.

Outpatients who met our inclusion criteria received an invitation for an epidemiological survey and a free personality assessment. The questionnaire and face-to-face interview were used as a two-stage process for diagnosing PDs. Individuals whose Personality Diagnostic Questionnaire Fourth Edition Plus (PDQ-4+)^16^ test results were positive (total score, >28 or specific PD subscale scores, >4 or 5) entered the second stage. Participants were referred to two senior psychiatrists with 5 years of experience; each received 2 weeks of training to perform the structured clinical interview for PD assessment. The final sample included in this analysis are 2090 patients whose self-reported questionnaires and face-to-face interview were completed.

### Measures

#### Demographic data

A self-made demographic questionnaire was administered to assess the participants’ personal, family, and social background including their physical and mental health conditions. (Table 1).

**Table 1:** Demographic characteristics, personality disorder traits and childhood traumatic experience, comparison of groups among clinical diagnoses.

Variables	In total	Schizophrenia	Mood Disorder	Anxiety Disorder	Comparison *F/χ*^2^	*p* value
Cases [n(%)]	2090	592 (28.3)	581 (27.8)	515 (24.6)	—	—
Age(years) [*Mean* (*SD*)]	30.6 (9.7)	30.1 (9.3)	31.6 (10.0)	30.5 (9.5)	*F* = 3.570	0.028
Male [*n*(*%*)]	932 (44.6)	257 (43.4)	224 (38.6)	256 (49.7)	*χ*^2^ = 13.832	0.001
Education level (≤9 years) [n(%)]	411 (19.7)	116 (19.6)	103 (17.7)	105 (20.4)	*χ*^2^ = 1.340	0.512
Married [*n*(*%*)]	802 (38.4)	149 (37.1)	250 (43.0)	245 (47.6)	*χ*^2^ = 67.518	<0.001
Have religion [*n*(*%*)]	531 (25.4)	170 (28.7)	146 (25.1)	125 (24.3)	*χ*^2^ = 3.274	0.195
Parental separation [*n*(*%*)]	192 (9.2)	45 (7.6)	55 (9.5)	43 (8.3)	*χ*^2^ = 1.330	0.514
First visit [*n*(*%*)]	1163 (55.6)	172 (29.1)	359 (61.8)	344 (66.8)	*χ*^2^ = 192.293	<0.001
Course of disease(months) [*Mean* (*SD*)]	57.5 (74.7)	76.4 (86.9)	49.1 (70.6)	45.3 (60.7)	*F* = 23.048	<0.001
Family History [*n*(*%*)]	236 (11.3)	70 (11.8)	70 (12.0)	58 (11.3)	*χ*^2^ = 0.171	0.918
**PD Diagnosis by SCID-II** (**Structured Clinical Interview for DSM-IV axis-II)**						
Paranoid [n(%)]	183 (8.8)	77 (13.0)	53 (9.1)	33 (6.4)	*χ*^2^ = 14.037	0.001
Schizoid [n(%)]	82 (3.9)	21 (3.5)	24 (4.1)	18 (3.5)	*χ*^2^ = 0.394	0.821
Schizotypal [n(%)]	87 (4.2)	40 (6.8)	17 (2.9)	16 (3.1)	*χ*^2^ = 13.056	0.001
Histrionic [n(%)]	70 (3.3)	11 (1.9)	18 (3.1)	26 (5.0)	*χ*^2^ = 8.966	0.011
Narcissistic [n(%)]	83 (4.0)	13 (2.2)	21 (3.6)	23 (4.5)	*χ*^2^ = 4.503	0.105
Borderline [n(%)]	178 (8.5)	23 (3.9)	83 (14.3)	34 (6.6)	*χ*^2^ = 44.492	<0.001
Antisocial [n(%)]	18 (0.9)	2 (0.3)	1 (0.2)	5 (1.0)	*χ*^2^ = 4.051	0.132
Avoidant [n(%)]	248 (11.9)	68 (11.5)	81 (13.9)	64 (12.4)	*χ*^2^ = 1.627	0.443
Dependent [n(%)]	91 (4.4)	32 (5.4)	26 (4.5)	21 (4.1)	*χ*^2^ = 1.172	0.557
Obsessive-compulsive [n(%)]	232 (11.1)	40 (6.8)	73 (12.6)	86 (16.7)	*χ*^2^ = 26.691	<0.001
Depressive [n(%)]	260 (12.4)	52 (8.8)	110 (18.9)	58 (11.3)	*χ*^2^ = 28.698	<0.001
Passive-aggressive [n(%)]	112 (5.4)	25 (4.2)	42 (7.2)	18 (3.5)	*χ*^2^ = 9.218	0.010
**PD traits by PDQ**-**4+ [Median, Mean** (**SD)]**						
Paranoid [Median, Mean (SD)]	3, 3.4 (1.7)	3, 3.3 (1.7)	3, 3.4 (1.7)	3, 3.4 (1.8)	*χ*^2^ = 1.604	0.448
Schizoid [Median, Mean (SD)]	2, 2.7 (1.5)	2, 2.7 (1.4)	3, 2.8 (1.6)	2, 2.6 (1.6)	*χ*^2^ = 2.050	0.359
Schizotypal [Median, Mean (SD)]	4, 4.3 (1.9)	4, 4.5 (1.9)	4, 4.3 (2.0)	4, 4.2 (1.9)	*χ*^2^ = 9.984	0.007
Histrionic [Median, Mean (SD)]	4, 4.0 (1.8)	4, 3.8 (1.9)	4, 4.1 (1.7)	4, 4.0 (1.7)	*χ*^2^ = 6.000	0.050
Narcissistic [Median, Mean (SD)]	4, 3.9 (2.0)	3, 3.7 (2.0)	4, 4.1 (2.0)	4, 3.8 (1.9)	*χ*^2^ = 13.227	0.001
Borderline [Median, Mean (SD)]	5, 5.0 (2.2)	4, 4.8 (2.3)	5, 5.4 (2.2)	5, 4.9 (2.0)	*χ*^2^ = 27.820	<0.001
Antisocial [Median, Mean (SD)]	2, 2.0 (1.7)	2, 2.1 (1.6)	2, 2.0 (1.7)	1, 1.8 (1.6)	*χ*^2^ = 17.432	<0.001
Avoidant [Median, Mean (SD)]	5, 4.3 (1.7)	4, 4.2 (1.7)	4, 4.6 (1.7)	5, 4.3 (1.8)	*χ*^2^ = 19.022	<0.001
Dependent [Median, Mean (SD)]	4, 3.8 (1.9)	4, 3.9 (1.9)	4, 4.1 (2.0)	4, 3.8 (1.9)	*χ*^2^ = 5.395	0.067
Obsessive-compulsive [Median, Mean (SD)]	4, 4.3 (1.8)	4, 4.2 (1.8)	5, 4.5 (1.7)	4, 4.4 (1.7)	*χ*^2^ = 8.093	0.017
Depressive [Median, Mean (SD)]	4, 4.3 (1.8)	4, 4.0 (1.9)	5, 4.6 (1.7)	4, 4.3 (1.8)	*χ*^2^ = 35.034	<0.001
Passive-aggressive [Median, Mean (SD)]	3, 3.3 (1.6)	3, 3.3 (1.6)	3, 3.5 (1.5)	3, 3.2 (1.5)	*χ*^2^ = 5.780	0.056
Total score of PD traits [Median, Mean (SD)]	44, 45.3 (13.5)	43, 44.4 (14.5)	47, 47.3 (13.7)	44, 44.5 (12.1)	*χ*^2^ = 16.241	<0.001
**Childhood traumatic experience by CTQ [Median, Mean** (**SD)]**						
Emotional abuse [Median, Mean (SD)]	7, 7.7 (3.2)	7, 7.8 (3.2)	7, 7.9 (3.4)	6, 7.5 (3.0)	*χ*^2^ = 5.948	0.051
Physical abuse [Median, Mean (SD)]	5, 6.3 (2.4)	5, 6.4 (2.4)	5, 6.3 (2.4)	5, 6.2 (2.3)	*χ*^2^ = 5.005	0.082
Sexual abuse [Median, Mean (SD)]	5, 5.9 (1.9)	5, 6.1 (2.1)	5, 5.8 (2.0)	5, 5.6 (1.4)	*χ*^2^ = 26.539	<0.001
Emotional neglect [Median, Mean (SD)]	18, 18.6 (4.8)	18, 18.3 (4.7)	18, 18.8 (5.1)	18, 18.5 (4.7)	*χ*^2^ = 2.232	0.328
Physical neglect [Median, Mean (SD)]	9, 9.1 (3.1)	9, 9.3 (3.0)	9, 8.9 (3.1)	9, 8.8 (3.1)	*χ*^2^ = 9.601	0.008
Total scores of abuse and neglect [Median, Mean (SD)]	46, 47.5 (10.6)	46, 47.9 (10.3)	45, 47.7 (11.1)	45, 46.5 (10.2)	*χ*^2^ = 6.293	0.043

#### PD trait

The PDQ-4+, a self-reported questionnaire, was used for assessing PD trait. Our previous study^8^ and other research has validated that the PDQ-4+ has a high sensitivity (0.89) and moderate specificity (0.65) for screening PD patients. The PDQ-4+ was designed to measure all 10 PDs traits in DSM-IV, including negativistic PD and depressive PD which are included in the Appendix of DSM-IV. Those 10 PD traits are Paranoid PD trait (7 items, cut-off = 4), Schizoid PD trait (7 items, cut-off = 4), Schizotypal PD trait (9 items, cut-off = 5), Histrionic PD trait (8 items, cut-off = 5), Narcissistic PD trait (9 items, cut-off = 5), Borderline PD trait (9 items, cut-off = 5), Antisocial PD trait (8 items, cut-off = 4), Avoidant PD trait (7 items, cut-off = 4), Dependent PD trait (8 items, cut-off = 5), Obsessive-compulsive PD trait (8 items, cut-off = 4).

#### PD diagnosis

The Structured Clinical Interview for DSM-IV Axis II (SCID-II)^17^ was designed to measure all 10 PDs in the DSM-IV, the criteria used for PD diagnoses in this study. The Chinese version of SCID-II was translated and implemented by our team. Previous studies have demonstrated that the SCID-II Chinese version has a relatively high test–retest reliability of 0.70, with a median coefficient for internal consistency of 0.70, which is highly consistent (diagnostic agreement rate of 90.7%) with the clinical diagnoses.

#### Childhood trauma experience (CTE)

A quantitative index of childhood adversity severity was assessed using the Child Trauma Questionnaire Short Form (CTQ-SF)^14,18^. The CTQ-SF consists of 5 CTE subscales from 28 self-reported items: emotional abuse, physical abuse, sexual abuse, emotional neglect, and physical neglect, with a range of 5 (low level of CTE) to 25 (high level of CTE).

### Statistical analysis

#### Correlation

**Figure 1:**
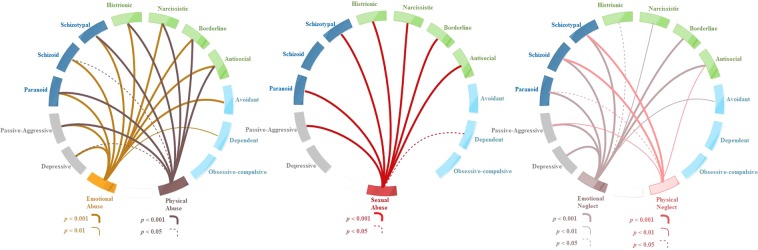
Spearman correlation between childhood traumatic experience(CTE) and personality disorder(PD) traits. Note: The connections between CTE and PD traits are sized based on the *p*-values.

**Figure 2:**
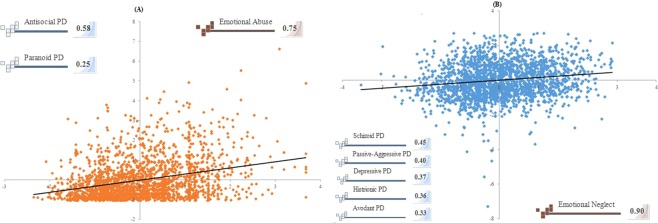
Two pairs (**A,B**) of canonical correlations between childhood traumatic experience (CTE) and personality disorder (PD) traits.

Relationships between PD traits and CTE were investigated using nonparametric correlations and CCA. Spearman-rank correlations were used to test for associations between PD traits (12 items) and CTE (5 items), comprising 60 combinations, in this case resulting in 43 significant correlations (Fig. 1). We used Spearman-rank correlations because clinical variables did not fit a normal, continuous distribution. For the purpose of selecting a subset of relevant, non-redundant clinical and cognitive features, CCA was applied to identify a representation of PD trait domains that were associated with weighted combinations of CTE. CCA determines pairs of linear combinations, termed canonical variables, from two sets of variables (PD trait and CTE) (Fig. 2), such that the correlation between canonical variables is maximized.

#### Classification

**Figure 3:**
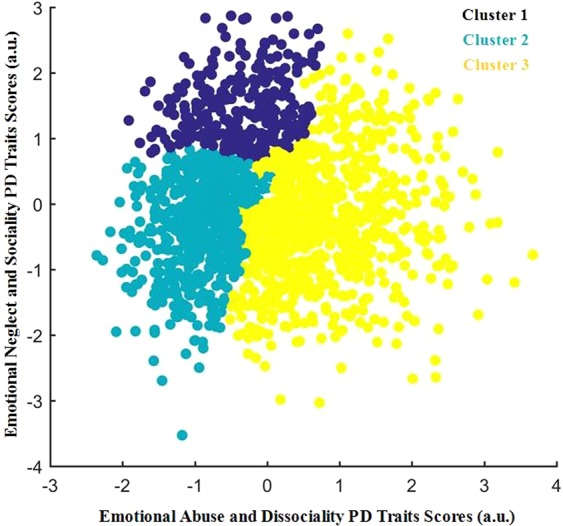
Hierarchical cluster analysis of two canonical variates and a scatterplot for the three clusters.

To find clusters in this 2-dimensional space of data points (each point represents an individual case), hierarchical cluster analysis was applied by using MATLAB’s pdist, linkage, cluster, and cluster data functions. For the current analysis, an average-linkage algorithm was used to cluster outpatients. Euclidean distance was used as a metric to evaluate sample similarity. This method was used in order to find clusters among the data points according to the inter-point and inter-cluster distances. The Euclidean distance between every pair of subjects in this 2-dimensional feature space was calculated, and then Ward’s minimum variance method was used to select a specific clustering from the dendrogram (Fig. 3), iteratively linking pairs of subjects in closest proximity, forming progressively larger clusters in a hierarchical tree. The hierarchical clustering analysis was used to delineate clusters of subjects in a two-dimensional space defined by these two canonical variates. This three-cluster solution was optimal for summarizing relatively homogeneous subgroups that were maximally dissimilar from each other. Additional potential clustering solutions, indicating four or five clusters, were also evident, nested within these subgroups. Detailed baseline demographic, PD diagnosis, PD traits and CTE for the three clusters can be found in the Table 2.

**Table 2:** Demographic and clinical characteristics, personality disorder traits and childhood traumatic experience, comparison of groups among three clusters.

Variables	Cluster 1	Cluster 2	Cluster 3	Comparison *F/χ*^2^	*p* value
Cases [n(%)]	365 (17.5)	727 (34.8)	998 (47.8)	—	—
Age (years) [*Mean* (*SD*)]	31.7 (10.3)	31.3 (9.7)	29.6 (9.3)	*F* = 9.894	<0.001
Male [*n*(*%*)]	158 (43.3)	304 (41.8)	470 (47.1)	*χ*^2^ = 5.048	0.080
Education level (≤9 years) [n(%)]	73 (20.0)	131 (18.0)	207 (20.7)	*χ*^2^ = 2.004	0.367
Married [*n*(*%*)]	152 (41.6)	297 (40.9)	353 (35.4)	*χ*^2^ = 7.346	0.025
Have religion [*n*(*%*)]	89 (24.4)	180 (24.8)	262 (26.3)	*χ*^2^ = 0.739	0.691
Parental separation [*n*(*%*)]	30 (8.2)	63 (8.7)	99 (9.9)	*χ*^2^ = 1.289	0.525
First visit [*n*(*%*)]	228 (62.5)	376 (51.7)	559 (56.0)	*χ*^2^ = 11.474	0.003
Course of disease (months) [*Mean* (*SD*)]	59.6 (68.5)	56.4 (78.0)	57.6 (74.4)	*F* = 0.159	0.853
Family History [*n*(*%*)]	38 (10.4)	78 (10.7)	120 (12.0)	*χ*^2^ = 1.047	0.593
**Clinical Diagnosis by SCID-I** (**Structured Clinical Interview for DSM-IV axis-I)**					
Schizophrenia [*n*(*%*)]	93 (25.5)	192 (26.4)	307 (30.8)	*χ*^2^ = 5.687	0.058
Mood Disorder [*n*(*%*)]	98 (26.8)	198 (27.2)	285 (28.6)	*χ*^2^ = 0.565	0.754
Anxiety Disorder [*n*(*%*)]	101 (27.7)	202 (27.8)	212 (21.2)	*χ*^2^ = 11.883	0.003
**PD Diagnosis by SCID-II** (**Structured Clinical Interview for DSM-IV axis-II)**					
Paranoid [n(%)]	27 (7.4)	37 (5.1)	119 (11.9)	*χ*^2^ = 25.613	<0.001
Schizoid [n(%)]	17 (4.7)	18 (2.5)	47 (4.7)	*χ*^2^ = 6.198	0.045
Schizotypal [n(%)]	21 (5.8)	13 (1.8)	53 (5.3)	*χ*^2^ = 15.887	<0.001
Histrionic [n(%)]	6 (1.6)	22 (3.0)	42 (4.2)	*χ*^2^ = 5.790	0.055
Narcissistic [n(%)]	13 (3.6)	13 (1.8)	57 (5.7)	*χ*^2^ = 17.171	<0.001
Borderline [n(%)]	14 (3.8)	33 (4.5)	131 (13.1)	*χ*^2^ = 52.244	<0.001
Antisocial [n(%)]	2 (0.5)	1 (0.1)	15 (1.5)	*χ*^2^ = 9.693	0.008
Avoidant [n(%)]	64 (17.5)	63 (8.7)	121 (12.1)	*χ*^2^ = 18.397	<0.001
Dependent [n(%)]	15 (4.1)	27 (3.7)	49 (4.9)	*χ*^2^ = 1.508	0.470
Obsessive-compulsive [n(%)]	46 (12.6)	75 (10.3)	111 (11.1)	*χ*^2^ = 1.288	0.525
Depressive [n(%)]	47 (12.9)	69 (9.5)	144 (14.4)	*χ*^2^ = 9.492	0.009
Passive-aggressive [n(%)]	21 (5.8)	17 (2.3)	74 (7.4)	*χ*^2^ = 21.508	<0.001
**PD traits by PDQ**-**4+ [Median, Mean** (**SD)]**					
Paranoid [Median, Mean (SD)]	3, 3.0 (1.6)	2, 2.4 (1.3)	5, 4.3 (1.6)	*F* = 358.696	<0.001
Schizoid [Median, Mean (SD)]	4, 3.5 (1.5)	2, 2.0 (1.2)	3, 2.9 (1.6)	*F* = 156.177	<0.001
Schizotypal [Median, Mean (SD)]	4, 4.4 (1.7)	3, 3.0 (1.4)	5, 5.2 (1.8)	*F* = 389.630	<0.001
Histrionic [Median, Mean (SD)]	4, 4.0 (1.7)	3, 3.2 (1.7)	5, 4.6 (1.6)	*F* = 146.913	<0.001
Narcissistic [Median, Mean (SD)]	3, 3.4 (1.7)	3, 2.8 (1.7)	5, 4.8 (1.8)	*F* = 289.860	<0.001
Borderline [Median, Mean (SD)]	4, 4.6 (1.8)	3, 3.5 (1.7)	6, 6.2 (2.0)	*F* = 472.994	<0.001
Antisocial [Median, Mean (SD)]	1, 0.9 (0.9)	1, 0.8 (0.8)	3, 3.3 (1.4)	*F* = 1211.412	<0.001
Avoidant [Median, Mean (SD)]	5, 4.7 (1.7)	4, 3.8 (1.6)	5, 4.6 (1.7)	*F* = 49.261	<0.001
Dependent [Median, Mean (SD)]	2, 2.6 (1.8)	4, 3.6 (1.7)	5, 4.5 (1.9)	*F* = 154.101	<0.001
Obsessive-compulsive [Median, Mean (SD)]	5, 4.5 (1.7)	4, 3.9 (1.8)	5, 4.6 (1.7)	*F* = 35.941	<0.001
Depressive [Median, Mean (SD)]	5, 5.1 (1.4)	3, 3.3 (1.8)	5, 4.6 (1.7)	*F* = 178.644	<0.001
Passive-aggressive [Median, Mean (SD)]	3, 3.4 (1.4)	2, 2.3 (1.2)	4, 4.0 (1.4)	*F* = 329.136	<0.001
Total score of PD traits [Median, Mean (SD)]	43, 44.1 (9.9)	33, 34.5 (8.9)	54, 53.5 (11.7)	*F* = 691.162	<0.001
**Childhood traumatic experience by CTQ [Median, Mean** (**SD)]**					
Emotional abuse [Median, Mean (SD)]	7, 7.3 (2.8)	6, 6.9 (2.5)	8, 8.5 (3.6)	*F* = 58.686	<0.001
Physical abuse [Median, Mean (SD)]	5, 6.1 (2.1)	5, 5.9 (2.0)	5, 6.7 (2.7)	*F* = 23.778	<0.001
Sexual abuse [Median, Mean (SD)]	5, 5.6 (1.4)	5, 5.6 (1.5)	5, 6.2 (2.3)	*F* = 26.360	<0.001
Emotional neglect [Median, Mean (SD)]	19, 19.3 (4.8)	17, 17.7 (4.6)	18, 18.9 (5.0)	*F* = 18.494	<0.001
Physical neglect [Median, Mean (SD)]	9, 9.1 (3.1)	9, 8.7 (2.9)	9, 9.3 (3.2)	*F* = 7.673	<0.001
Total scores of abuse and neglect [Median, Mean (SD)]	46, 47.4 (9.5)	43, 44.8 (9.0)	47, 49.5 (11.5)	*F* = 44.028	<0.001

#### Validation

**Figure 4:**
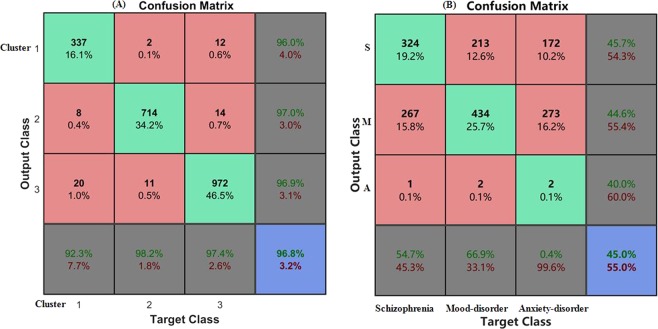
Support Vector Machine(SVM) model for three-cluster solution (**A**) and clinical diagnostic classifications (**B**).

We further assessed the utility of the extracted sub-clusters of outpatients in classification clinical diagnosis. To evaluate whether subtypes are identical to clinical phenomenological diagnostic definitions, Support Vector Machine (SVM) model was trained and validated using the Linear kernel to illustrate the relationship of this three-cluster solution to clinical diagnostic classifications (Fig. 4).

## Results

### Sample characteristics

Characteristics of 2090 patients are presented in Table 1, including demographics, clinical diagnosis, PD diagnosis, PDQ-4+ scores, and CTQ scores.

### Correlations

Most CTQ scores were significantly associated with PD traits (Fig. 1), especially the relationship between emotional abuse and Clusters A and B PD traits, physical abuse and Cluster B PD traits, sexual abuse and Cluster B PD traits, and emotional/physical neglect and Cluster A PD traits.

### Canonical correlation

The CCA identified a dimensional representation of CTE, quantified by the CTQ, that was associated with the dimensional representation of PD traits, quantified by the PDQ-4+. After performing the CCA, we determined two linear combinations of PD trait (canonical variates) that were correlated with distinct CTE combinations, which we term “emotion abuse-related dissociality PD traits” (Fig. 2A) and “emotion neglect-related sociality PD traits” (Fig. 2B).

The first leading pair of dimensions extracted by the CCA from CTE and PD traits showed a statistically significant correlation coefficient of 0.31 (*p* < 0.001). The first CTE components (canonical variates) indicated that primarily emotional abuse was correlated with antisocial and paranoid PD traits (Fig. 2A), which were defined as dissociality PD traits. The scatterplot illustrates the correlation between dimensional CTQ scores and dimensional clinical scores for the PDQ-4+. To the left of scatterplot, PDQ-4+ score loadings are depicted for those PD traits with the strongest loadings. To the right of the scatterplot, CTQ score loadings are depicted for emotional abuse, which demonstrated the strongest loading.

The second leading pair of dimensions extracted from the CCA showed a strong tendency towards a statistically significant correlation coefficient of 0.12 (*p* < 0.001). The second CTE component indicated that primarily emotional neglect was correlated with schizoid, passive-aggressive, depressive, histrionic, and avoidant PD traits (Fig. 2B), which was defined to be sociality PD traits.

### Hierarchical cluster analysis

To explore clusters, a hierarchical cluster analysis was applied to assign samples to nested subgroups with similar patterns of relationships between CTE and PD traits. Our analysis revealed three clusters defined by distinct and relatively homogeneous patterns along two dimensions (Fig. 3), comprising 17.5% (cluster 1, n = 365), 34.8% (cluster 2, n = 727), and 47.8% (cluster 3, n = 998) of the 2090 participant sample.

### Validation

The three-cluster SVM model was trained and validated using the linear kernel. The results of the full analysis (confusion matrix, accuracy, sensitivity, and specificity) are presented in Fig. 4. Figure 4A shows the confusion matrix for the three clusters obtained by SVMs with linear kernel, achieving an overall accuracy of 96.8%. However, Fig. 4B shows the three clinical diagnostic categories (schizophrenia, mood disorder, anxiety disorder) only achieved an overall accuracy of 45.0%.

### Distribution of subtypes across clinical diagnoses

**Figure 5:**
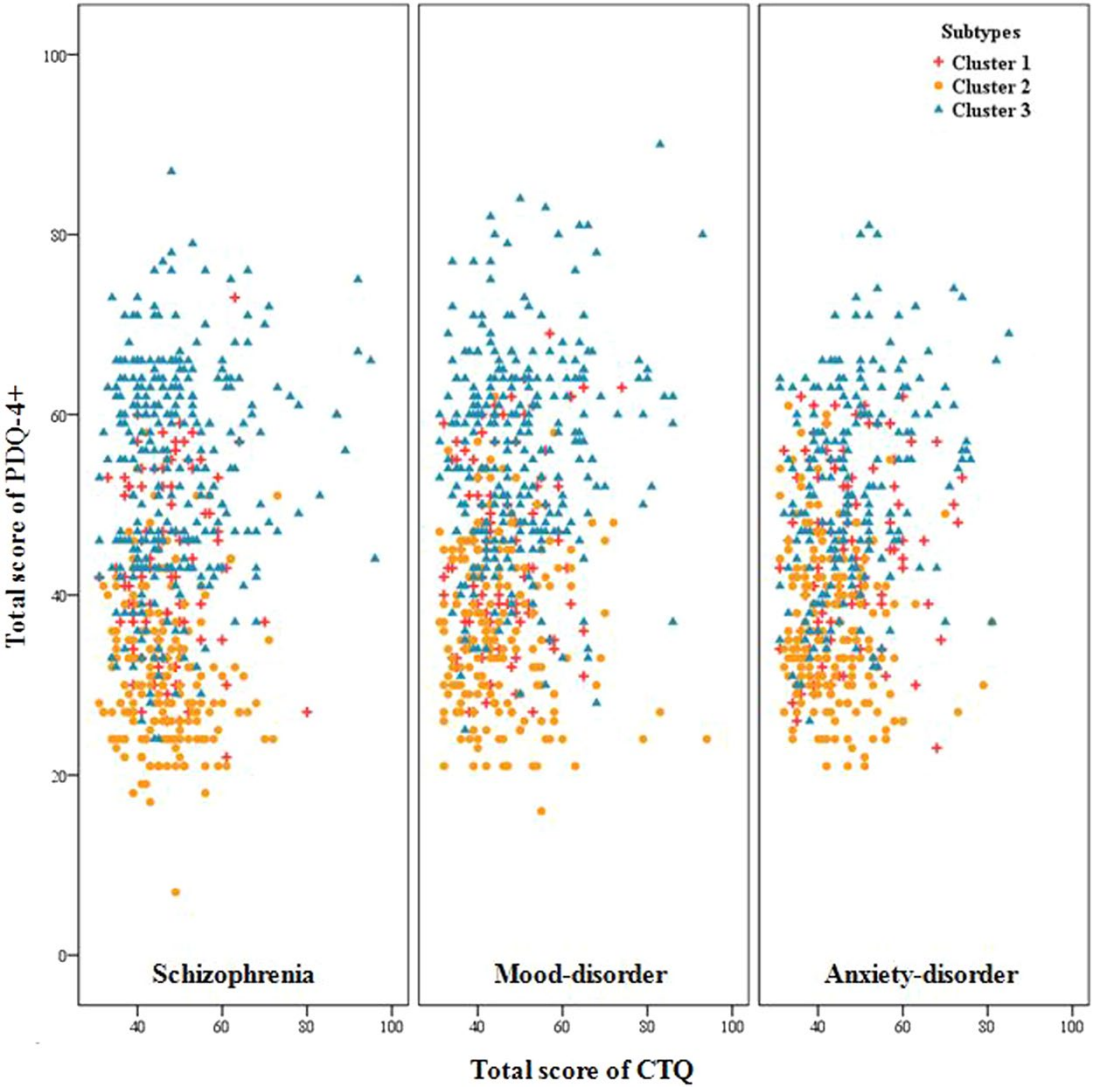
Distribution of personality disorder (PD) traits and childhood traumatic experience (CTE) scores by subtype and clinical diagnosis.

Table 2 depicts the demographic, clinical Axis-I diagnosis, PD diagnosis, and CTE profiles of the three clusters defined by the CCA. The features of the three subtypes are summarized as follows: Cluster 3 had the highest proportion of PD, and reported the most severe pathology of PD traits, and the most severe CTE. Cluster 1 was moderately impaired on PD traits and reported moderate CTE. Cluster 2 had the lowest proportion of PD, and reported the mildest pathology of PD traits, and the least CTE. As illustrated in Fig. 5, there was considerable mixing across subtypes of clinical diagnoses. Similarly, there was considerable overlap across the clinical diagnoses on PD traits and CTE scores. There was an equal distribution of clinical diagnoses across subtypes (Table 2), except for anxiety disorders. These subtypes suggest more distinct PD trait correlates of CTE manifestation than were captured by clinical phenomenological diagnostic definitions.

## Discussion

The present study used CCA to determine whether psychiatric patients display different subtypes that can be distinguished by combinations of PD traits and CTE variables. We found three such subtypes independent of specific clinical diagnostic classifications. Each subtype included all psychiatric categories, but in Cluster 3, there were higher and lower numbers of patients with schizophrenia and anxiety disorders, respectively.

An important feature of our CCA approach was that multicollinearity (highly correlated relationship between the variables make it difficult for the model to estimate accurately) was avoided^19^. CCA uses information from all the variables in the PD traits and CTE variable sets, maximizes the estimation of the relationship between the two sets^20^, and may identify subtypes in a more efficient way. Compared with conventional multiple testing, CCA may also help reduce type 1 error and increase result accuracy. Our analyses produced statistical support for the existence of three subtypes within the Chinese psychiatric patients, as well as effective characteristic variables for distinguishing Cluster 3 patients from the other two clusters, which contained the most severe pathology of personality traits.

The present findings provide the first evidence, to our knowledge, of transdiagnostic subtypes through the correlation between PD traits and CTE at the clinical population level. This result is linked to specific units of an RDoC analysis of self-reports^21^ and stands in contrast to other RDoC studies that have identified subtypes via biological dimensions^22–24^. Cluster 3 was characterized by extensive and severe dissociality PD traits and more emotional abuse during childhood. In contrast, individuals in Cluster 1 were characterized by severe sociality PD traits and experienced more emotional neglect. Individuals in Cluster 2 showed a lesser severity of PD traits and CTE. These differential patterns of PD traits and CTE across subtypes invoke an explanation for the marked diagnostic disagreement^25^ in psychiatric disorders that is routinely observed across clinicians^26^. Our study followed with the RDoC approach suggest that transdiagnostic factors such as PD traits and CTE that may be affect clinical classification of subtypes, rather than by the current overreliance of the classification of patients to psychiatric diagnosis.

These data also indicate that there may be multiple pathways to similar clinically psychiatric manifestations. Subtypes identified in the present study were also distributed fairly uniformly across all clinical categories, especially in affective disorders, suggesting that PD traits and CTE^27^ have broadband transdiagnostic associations across common psychiatric disorders^28^. This finding extends the results of previous RDoC studies^29,30^ to the personality pathology-childhood adversity dimensions of a wide range of disorders. Furthermore, by exploring CTE and its association with PD traits, the present study was able to examine the role of differential types of CTE in PD traits in a transdiagnostic framework.

### Clinical implications

The heterogeneity of psychiatric diagnosis has emerged as a major obstacle to the development of intervention strategies and, in particular, methods of psychotherapy targeting personality^31^ and trauma^32^. Similarly, efforts to validate and replicate certain psychotherapies are inefficient, owing in part to a lack of ability to select psychiatric patients for intervention, who are most likely to benefit from personality remodelling and psychological trauma rehabilitation. Further, psychiatric disorders are treated as a single label for heterogeneous PD traits and CTE, which is problematic as it is clear that varying subtypes should be treated differently. In the current study, we determined three subtypes of patients that were associated with different patterns of PD traits and distinct CTE, which have also been broadly distributed across psychiatric diagnoses. However, we are in the initial stages of this subtype approach; therefore, it is premature to suggest definitive claims until more clinical interventions targeting subtypes of patients are conducted.

### Strengths and limitations

The strengths of the present study include its novelty in developing CCA-driven subtypes for a clinical population, based on a large-scale, transdiagnostic, psychiatric sample. The use of canonical variates (i.e., combination of PD traits and CTE) as indicators is novel. However, several limitations of this study must be considered. First, a single centre was used for sample recruitment, which could limit our ability to generalize the findings. However, the centre is the largest psychiatric hospital in China, which serves over 800,000 outpatients per year with more than half coming from across the nation. Second, this is a cross-sectional design; it remains unknown whether the three subtypes of patients predicted symptom onset and maintenance. Future longitudinal studies in the experimental manipulation of psychotherapy will be important for clarifying the precise role of these subtypes. Third, CTE was measured based on retrospective self-reports, which can be biased^33^ and may be overestimated in patients with negative life outcomes in adulthood^34^. Fourth, the current dataset was only included for those whose PDQ-4+ screening was positive, which could result in uncertain whether there are any other subtypes can be identified among patients whose PDQ-4+ test was negative. Finally, while the current dataset contains a relatively large number of sample, it is limited by the single site design without independent sample for the model validation. Thus, the validation by SVM method for 3 clusters model may suffer from overfitting.

## Conclusion

The present study extends the previous work of the transdiagnostic approach by applying a CCA-driven approach to determine subtypes for capturing PD traits-CTE distinctiveness in psychiatric disorders. Three subtypes emerged with distinctive features based on two dimensions: emotional abuse-dissociality PD traits and emotional neglect-sociality PD traits. If replicated, findings would suggest the clinical utility of these subtypes in psychiatric practice. Further research is needed to explore whether psychiatric outcomes can be improved by more individualized (i.e., by subtype) psychosocial interventions.
